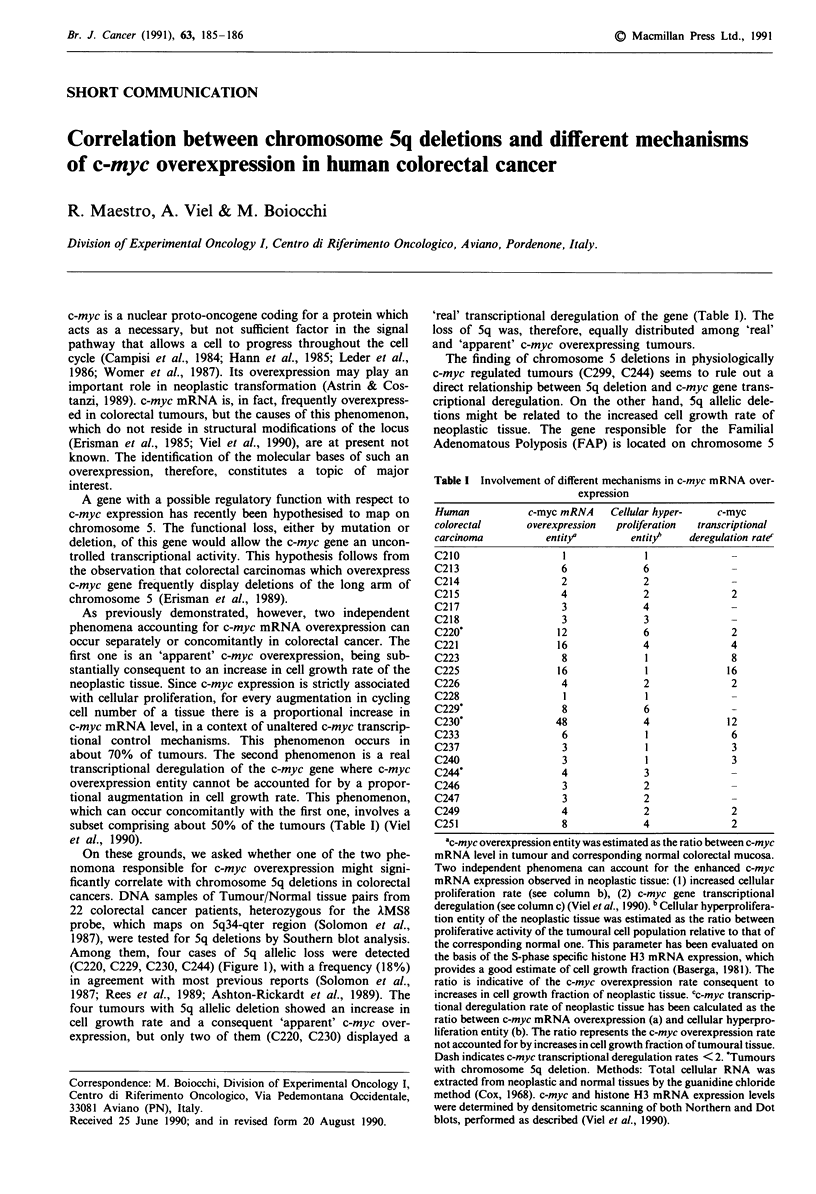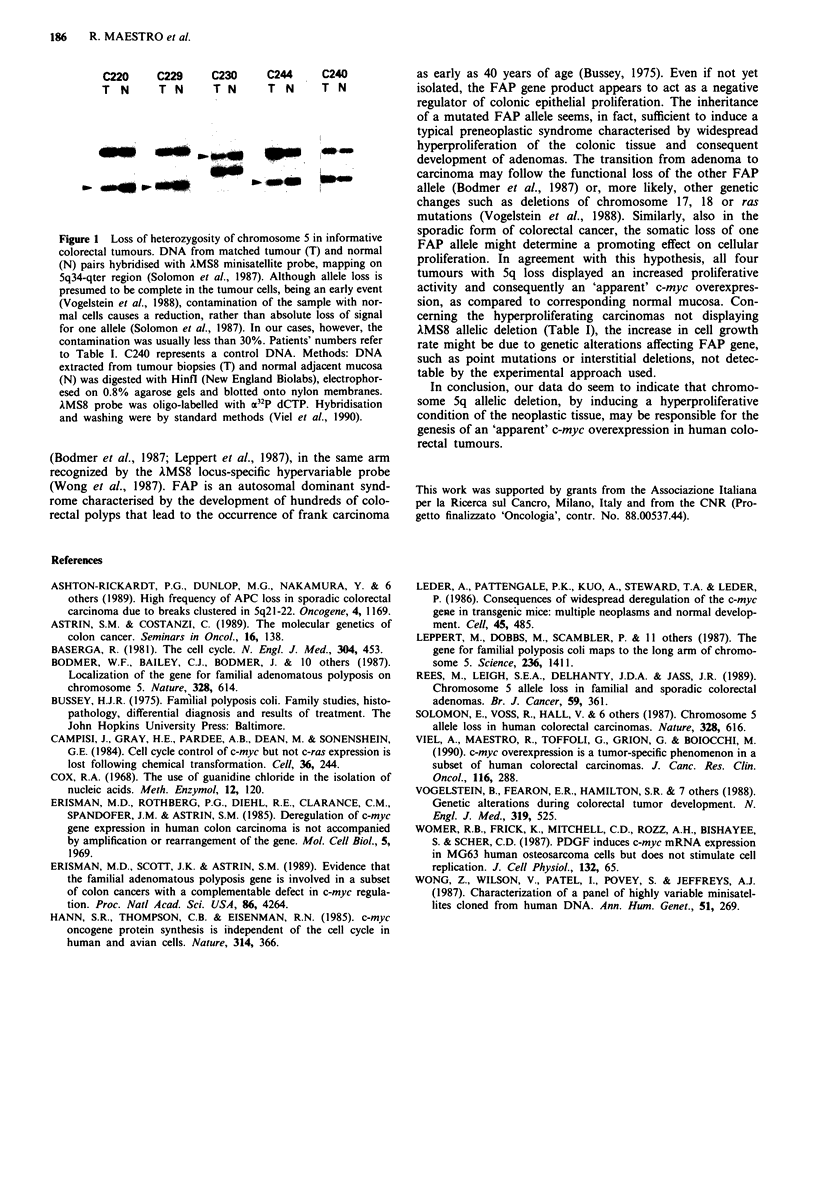# Correlation between chromosome 5q deletions and different mechanisms of c-myc overexpression in human colorectal cancer.

**DOI:** 10.1038/bjc.1991.45

**Published:** 1991-02

**Authors:** R. Maestro, A. Viel, M. Boiocchi

**Affiliations:** Division of Experimental Oncology I, Centro di Riferimento Oncologico, Aviano, Pordenone, Italy.

## Abstract

**Images:**


Br. J. Cancer (1991), 63, 185-186 © Macmillan Press Ltd., 1991

SHORT COMMUNICATION

### Correlation between chromosome 5q deletions and different mechanisms of c-*myc* overexpression in human colorectal cancer

R. Maestro, A. Viel & M. Boiocchi

*Division of Experimental Oncology I, Centro di Riferimento Oncologico, Aviano, Pordenone, Italy.*

c-*myc* is a nuclear proto-oncogene coding for a protein which acts as a necessary, but not sufficient factor in the signal pathway that allows a cell to progress throughout the cell cycle (Campisi *et al.*, 1984; Hann *et al.*, 1985; Leder *et al.*, 1986; Womer *et al.*, 1987). Its overexpression may play an important role in neoplastic transformation (Astrin & Costanzi, 1989). c-*myc* mRNA is, in fact, frequently overexpressed in colorectal tumours, but the causes of this phenomenon, which do not reside in structural modifications of the locus (Erisman *et al.*, 1985; Viel *et al.*, 1990), are at present not known. The identification of the molecular bases of such an overexpression, therefore, constitutes a topic of major interest.

A gene with a possible regulatory function with respect to c-*myc* expression has recently been hypothesised to map on chromosome 5. The functional loss, either by mutation or deletion, of this gene would allow the c-*myc* gene an uncontrolled transcriptional activity. This hypothesis follows from the observation that colorectal carcinomas which overexpress c-*myc* gene frequently display deletions of the long arm of chromosome 5 (Erisman *et al.*, 1989).

As previously demonstrated, however, two independent phenomena accounting for c-*myc* mRNA overexpression can occur separately or concomitantly in colorectal cancer. The first one is an 'apparent' c-*myc* overexpression, being substantially consequent to an increase in cell growth rate of the neoplastic tissue. Since c-*myc* expression is strictly associated with cellular proliferation, for every augmentation in cycling cell number of a tissue there is a proportional increase in c-*myc* mRNA level, in a context of unaltered c-*myc* transcriptional control mechanisms. This phenomenon occurs in about 70% of tumours. The second phenomenon is a real transcriptional deregulation of the c-*myc* gene where c-*myc* overexpression entity cannot be accounted for by a proportional augmentation in cell growth rate. This phenomenon, which can occur concomitantly with the first one, involves a subset comprising about 50% of the tumours (Table I) (Viel *et al.*, 1990).

On these grounds, we asked whether one of the two phenomona responsible for c-*myc* overexpression might significantly correlate with chromosome 5q deletions in colorectal cancers. DNA samples of Tumour/Normal tissue pairs from 22 colorectal cancer patients, heterozygous for the λMS8 probe, which maps on 5q34-qter region (Solomon *et al.*, 1987), were tested for 5q deletions by Southern blot analysis. Among them, four cases of 5q allelic loss were detected (C220, C229, C230, C244) (Figure 1), with a frequency (18%) in agreement with most previous reports (Solomon *et al.*, 1987; Rees *et al.*, 1989; Ashton-Rickardt *et al.*, 1989). The four tumours with 5q allelic deletion showed an increase in cell growth rate and a consequent 'apparent' c-*myc* overexpression, but only two of them (C220, C230) displayed a 'real' transcriptional deregulation of the gene (Table I). The loss of 5q was, therefore, equally distributed among 'real' and 'apparent' c-*myc* overexpressing tumours.

The finding of chromosome 5 deletions in physiologically c-*myc* regulated tumours (C299, C244) seems to rule out a direct relationship between 5q deletion and c-*myc* gene transcriptional deregulation. On the other hand, 5q allelic deletions might be related to the increased cell growth rate of neoplastic tissue. The gene responsible for the Familial Adenomatous Polyposis (FAP) is located on chromosome 5

**Table I** Involvement of different mechanisms in c-*myc* mRNA overexpression

| *Human colorectal carcinoma* | c-myc *mRNA overexpression entity*[a] | *Cellular hyper-proliferation entity*[b] | c-myc *transcriptional deregulation rate*[c] |
|---|---|---|---|
| C210 | 1 | 1 | – |
| C213 | 6 | 6 | – |
| C214 | 2 | 2 | – |
| C215 | 4 | 2 | 2 |
| C217 | 3 | 4 | – |
| C218 | 3 | 3 | – |
| C220* | 12 | 6 | 2 |
| C221 | 16 | 4 | 4 |
| C223 | 8 | 1 | 8 |
| C225 | 16 | 1 | 16 |
| C226 | 4 | 2 | 2 |
| C228 | 1 | 1 | – |
| C229* | 8 | 6 | – |
| C230* | 48 | 4 | 12 |
| C233 | 6 | 1 | 6 |
| C237 | 3 | 1 | 3 |
| C240 | 3 | 1 | 3 |
| C244* | 4 | 3 | – |
| C246 | 3 | 2 | – |
| C247 | 3 | 2 | – |
| C249 | 4 | 2 | 2 |
| C251 | 8 | 4 | 2 |

[a]c-*myc* overexpression entity was estimated as the ratio between c-*myc* mRNA level in tumour and corresponding normal colorectal mucosa. Two independent phenomena can account for the enhanced c-*myc* mRNA expression observed in neoplastic tissue: (1) increased cellular proliferation rate (see column b), (2) c-*myc* gene transcriptional deregulation (see column c) (Viel *et al.*, 1990). [b] Cellular hyperproliferation entity of the neoplastic tissue was estimated as the ratio between proliferative activity of the tumoural cell population relative to that of the corresponding normal one. This parameter has been evaluated on the basis of the S-phase specific histone H3 mRNA expression, which provides a good estimate of cell growth fraction (Baserga, 1981). The ratio is indicative of the c-*myc* overexpression rate consequent to increases in cell growth fraction of neoplastic tissue. [c]c-*myc* transcriptional deregulation rate of neoplastic tissue has been calculated as the ratio between c-*myc* mRNA overexpression (a) and cellular hyperproliferation entity (b). The ratio represents the c-*myc* overexpression rate not accounted for by increases in cell growth fraction of tumoural tissue. Dash indicates c-*myc* transcriptional deregulation rates $<2$. *Tumours with chromosome 5q deletion. Methods: Total cellular RNA was extracted from neoplastic and normal tissues by the guanidine chloride method (Cox, 1968). c-*myc* and histone H3 mRNA expression levels were determined by densitometric scanning of both Northern and Dot blots, performed as described (Viel *et al.*, 1990).

Correspondence: M. Boiocchi, Division of Experimental Oncology I, Centro di Riferimento Oncologico, Via Pedemontana Occidentale, 33081 Aviano (PN), Italy.
Received 25 June 1990; and in revised form 20 August 1990.

**Figure 1** Loss of heterozygosity of chromosome 5 in informative colorectal tumours. DNA from matched tumour (T) and normal (N) pairs hybridised with λMS8 minisatellite probe, mapping on 5q34-qter region (Solomon *et al.*, 1987). Although allele loss is presumed to be complete in the tumour cells, being an early event (Vogelstein *et al.*, 1988), contamination of the sample with normal cells causes a reduction, rather than absolute loss of signal for one allele (Solomon *et al.*, 1987). In our cases, however, the contamination was usually less than 30%. Patients' numbers refer to Table I. C240 represents a control DNA. Methods: DNA extracted from tumour biopsies (T) and normal adjacent mucosa (N) was digested with HinfI (New England Biolabs), electrophoresed on 0.8% agarose gels and blotted onto nylon membranes. λMS8 probe was oligo-labelled with $\alpha^{32}P$ dCTP. Hybridisation and washing were by standard methods (Viel *et al.*, 1990).

(Bodmer *et al.*, 1987; Leppert *et al.*, 1987), in the same arm recognized by the λMS8 locus-specific hypervariable probe (Wong *et al.*, 1987). FAP is an autosomal dominant syndrome characterised by the development of hundreds of colorectal polyps that lead to the occurrence of frank carcinoma as early as 40 years of age (Bussey, 1975). Even if not yet isolated, the FAP gene product appears to act as a negative regulator of colonic epithelial proliferation. The inheritance of a mutated FAP allele seems, in fact, sufficient to induce a typical preneoplastic syndrome characterised by widespread hyperproliferation of the colonic tissue and consequent development of adenomas. The transition from adenoma to carcinoma may follow the functional loss of the other FAP allele (Bodmer *et al.*, 1987) or, more likely, other genetic changes such as deletions of chromosome 17, 18 or *ras* mutations (Vogelstein *et al.*, 1988). Similarly, also in the sporadic form of colorectal cancer, the somatic loss of one FAP allele might determine a promoting effect on cellular proliferation. In agreement with this hypothesis, all four tumours with 5q loss displayed an increased proliferative activity and consequently an 'apparent' c-*myc* overexpression, as compared to corresponding normal mucosa. Concerning the hyperproliferating carcinomas not displaying λMS8 allelic deletion (Table I), the increase in cell growth rate might be due to genetic alterations affecting FAP gene, such as point mutations or interstitial deletions, not detectable by the experimental approach used.

In conclusion, our data do seem to indicate that chromosome 5q allelic deletion, by inducing a hyperproliferative condition of the neoplastic tissue, may be responsible for the genesis of an 'apparent' c-*myc* overexpression in human colorectal tumours.

This work was supported by grants from the Associazione Italiana per la Ricerca sul Cancro, Milano, Italy and from the CNR (Progetto finalizzato 'Oncologia', contr. No. 88.00537.44).

### References

ASHTON-RICKARDT, P.G., DUNLOP, M.G., NAKAMURA, Y. & 6 others (1989). High frequency of APC loss in sporadic colorectal carcinoma due to breaks clustered in 5q21-22. *Oncogene*, **4**, 1169.

ASTRIN, S.M. & COSTANZI, C. (1989). The molecular genetics of colon cancer. *Seminars in Oncol.*, **16**, 138.

BASERGA, R. (1981). The cell cycle. *N. Engl. J. Med.*, **304**, 453.

BODMER, W.F., BAILEY, C.J., BODMER, J. & 10 others (1987). Localization of the gene for familial adenomatous polyposis on chromosome 5. *Nature*, **328**, 614.

BUSSEY, H.J.R. (1975). Familial polyposis coli. Family studies, histopathology, differential diagnosis and results of treatment. The John Hopkins University Press: Baltimore.

CAMPISI, J., GRAY, H.E., PARDEE, A.B., DEAN, M. & SONENSHEIN, G.E. (1984). Cell cycle control of c-*myc* but not c-*ras* expression is lost following chemical transformation. *Cell*, **36**, 244.

COX, R.A. (1968). The use of guanidine chloride in the isolation of nucleic acids. *Meth. Enzymol*, **12**, 120.

ERISMAN, M.D., ROTHBERG, P.G., DIEHL, R.E., CLARANCE, C.M., SPANDOFER, J.M. & ASTRIN, S.M. (1985). Deregulation of c-*myc* gene expression in human colon carcinoma is not accompanied by amplification or rearrangement of the gene. *Mol. Cell Biol.*, **5**, 1969.

ERISMAN, M.D., SCOTT, J.K. & ASTRIN, S.M. (1989). Evidence that the familial adenomatous polyposis gene is involved in a subset of colon cancers with a complementable defect in c-*myc* regulation. *Proc. Natl Acad. Sci. USA*, **86**, 4264.

HANN, S.R., THOMPSON, C.B. & EISENMAN, R.N. (1985). c-*myc* oncogene protein synthesis is independent of the cell cycle in human and avian cells. *Nature*, **314**, 366.

LEDER, A., PATTENGALE, P.K., KUO, A., STEWARD, T.A. & LEDER, P. (1986). Consequences of widespread deregulation of the c-*myc* gene in transgenic mice: multiple neoplasms and normal development. *Cell*, **45**, 485.

LEPPERT, M., DOBBS, M., SCAMBLER, P. & 11 others (1987). The gene for familial polyposis coli maps to the long arm of chromosome 5. *Science*, **236**, 1411.

REES, M., LEIGH, S.E.A., DELHANTY, J.D.A. & JASS, J.R. (1989). Chromosome 5 allele loss in familial and sporadic colorectal adenomas. *Br. J. Cancer*, **59**, 361.

SOLOMON, E., VOSS, R., HALL, V. & 6 others (1987). Chromosome 5 allele loss in human colorectal carcinomas. *Nature*, **328**, 616.

VIEL, A., MAESTRO, R., TOFFOLI, G., GRION, G. & BOIOCCHI, M. (1990). c-*myc* overexpression is a tumor-specific phenomenon in a subset of human colorectal carcinomas. *J. Canc. Res. Clin. Oncol.*, **116**, 288.

VOGELSTEIN, B., FEARON, E.R., HAMILTON, S.R. & 7 others (1988). Genetic alterations during colorectal tumor development. *N. Engl. J. Med.*, **319**, 525.

WOMER, R.B., FRICK, K., MITCHELL, C.D., ROZZ, A.H., BISHAYEE, S. & SCHER, C.D. (1987). PDGF induces c-*myc* mRNA expression in MG63 human osteosarcoma cells but does not stimulate cell replication. *J. Cell Physiol.*, **132**, 65.

WONG, Z., WILSON, V., PATEL, I., POVEY, S. & JEFFREYS, A.J. (1987). Characterization of a panel of highly variable minisatellites cloned from human DNA. *Ann. Hum. Genet.*, **51**, 269.